# Factors modulating the effects of waking rest on memory

**DOI:** 10.1007/s10339-019-00942-x

**Published:** 2019-11-25

**Authors:** Markus Martini, Pierre Sachse

**Affiliations:** University of Innsbruck, Innrain 52, Innsbruck, Austria

**Keywords:** Waking rest, Wakeful resting, Interference, Consolidation, Memory

## Abstract

Study results indicate that moments of unoccupied rest immediately after learning serve an essential cognitive function: memory consolidation. However, there also are findings suggesting that waking rest after learning has similar effects on delayed memory performance as an active wake condition, where participants work on a cognitive distractor task. Based on these studies, we highlight several potentially modulating factors of the so-called resting effect.

In a highly relevant *Forum* article on the impact of a brief period of rest after learning on memory consolidation, Wamsley (2019) reviewed studies showing that memory performance at a later time point is higher under conditions where participants close their eyes and relax for several minutes subsequent to learning, compared to performing a cognitive distractor task (e.g. watching videos, listening to the radio, detecting tones; see Dewar et al. 2007; Fig. 1a). One assumption is that, in contrast to distraction after learning, waking rest supports the consolidation of previously learned memory content. Memory consolidation describes a family of neural processes responsible for stabilising and transforming memories such that they are accessible seconds, and even years, after their acquisition (Dudai et al. 2015). One proposed mechanism of memory consolidation is neural replay (Carr et al. 2011). Neural replay can be described as iterative reactivation of memory traces in the brain. Through this process, an initially labile memory content becomes stabilised, increasing the probability that this information can be recalled at a later time point (Dudai et al. 2015).

**Fig. 1:**
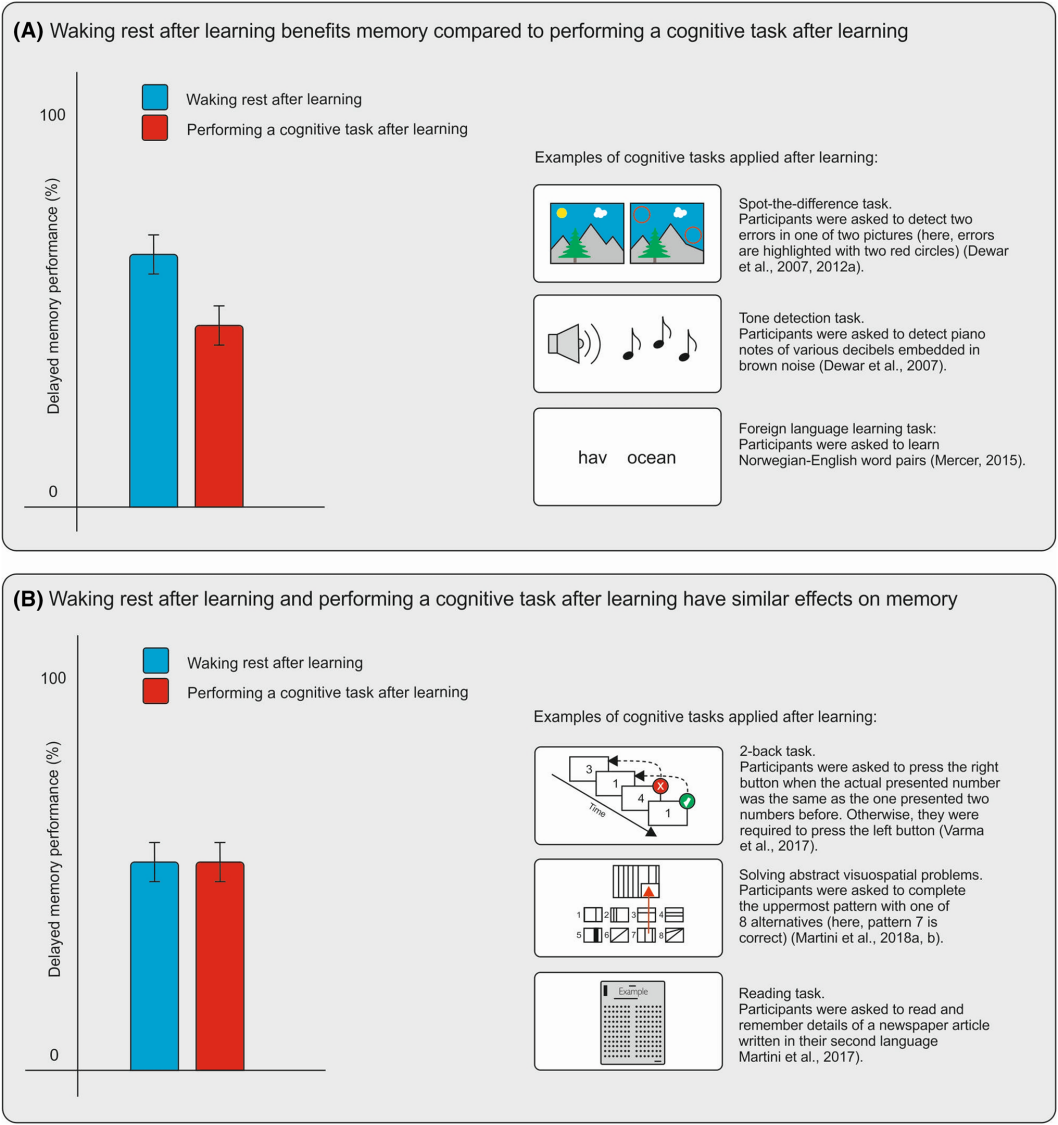
Illustrative depiction of hypothetical research outcomes when delayed memory performance is measured under a ‘waking rest after learning’ condition and a ‘cognitive task after learning’ condition. On the left side of (**a**), a hypothetical outcome is depicted showing that delayed memory performance is higher in the condition in which participants wakefully rest for several minutes after learning, compared to a condition in which participants perform a cognitive task for several minutes after learning. On the right side of (**a**), three exemplary tasks are presented from studies that found beneficial effects of waking rest compared to performing one of those tasks. On the left side of (**b)**, a hypothetical outcome is depicted when waking rest after learning and performing a cognitive task after learning have similar effects on delayed memory performance. On the right side of (**b**), three exemplary tasks are presented from studies that found no differences in delayed memory performance between the waking rest condition and the cognitive task condition

A host of different studies indicate that reduced interference after learning induced by, for instance, waking rest, slow-wave sleep, NMDA receptor antagonists, benzodiazepines, alcohol, and acetylcholine antagonists, facilitate memory consolidation (see Dudai et al. 2015; Mednick et al. 2011; Wamsley 2019), whereas transcranial magnetic stimulation, protein synthesis inhibitors, as well as task-related cognition immediately after learning can disrupt memory consolidation (Dewar et al. 2007; McGaugh 2015; Robertson 2012). The majority of waking rest studies, in which the impact of an ‘eyes closed-relaxed’ phase immediately after learning (waking rest condition) was tested against a ‘cognitive task’ phase immediately after learning (distractor condition), showed higher delayed memory performances in the waking rest condition (i) over shorter (minutes: Dewar et al. 2007) and longer (days: Dewar et al. 2012a) retention intervals; (ii) in different populations (children: Martini et al. 2019b; younger adults: Craig et al. 2015; older adults: Dewar et al. 2012a, b; amnesic patients: Dewar et al. 2009; patients with Alzheimer’s disease: Dewar et al. 2012b), (iii) with various learning materials (verbal: Dewar et al., 2012a, b; visuospatial: Craig et al. 2015), and (iv) post-learning cognitive distractor tasks (see Dewar et al. 2007; Mercer 2015; see Fig. 1a).

However, there is also evidence that brief periods of waking rest and distraction induced by performing a new task immediately after learning have similar effects on memory (consolidation) (Martini et al. 2017; Varma et al. 2017; Fig. 1b). Findings of these studies offer the opportunity to define potentially modulating factors of the resting effect: the memory consolidation supporting effect of a brief period of wakeful resting after learning compared to distraction after learning induced by performing a new cognitive task. In the following, we present a list of factors which were found to potentially modulate the resting effect (for a discussion of the role of intentional rehearsal during waking rest, see Wamsley 2019):

1. *Encoding and recall times*. Fatania and Mercer (2017; Experiment 1) showed a beneficial effect of a 5-min waking rest phase immediately after encoding a word list compared to working on spot-the-difference puzzles in a study with 6–7-year-old children. However, when learning and recall times were increased (Experiment 2), children showed similar delayed memory performances in the waking rest and spot-the-difference conditions. These results indicate that prolonging encoding and recall times can reverse the resting effect (see also Martini et al. (2018a)).
2. *Age*. Fatania and Mercer (2017; Experiment 1) showed no beneficial effect of 5-min waking rest in adults (18–61 years) but did find it in children aged 6–7 years. Martini et al. (2018b) found a beneficial effect of 8-min waking rest in older adults (60+ years) but not in younger adults (18–29 years), at least when the waking rest condition was followed by a distractor condition in which participants were required to solve abstract visuospatial problems (see also (7) *Order of the waking rest condition*). These results indicate that, under certain conditions, waking rest has a higher impact on memory consolidation in children and older adults compared to younger adults (but see also Craig et al. 2016).
3. *Cognitive tasks after learning.* In several experiments with younger adults, Varma and colleagues (2017) found similar effects of waking rest and verbal and visual 2-/3-back tasks on the retention of verbal and visual information. They argue that post-encoding cognitive engagement does not interfere with memory consolidation when task performance has minimal semantic and hippocampal-based episodic memory processing demands (see also Martini et al. 2017, 2018a; Experiment 2).
4. *Temporal position of the distractor task.* A waking rest study from Dewar et al. (2009) showed that amnesic patients’ memory performance was significantly higher when the temporal onset of the distractor task was delayed by 6 min (compared to a 3-min delay or no delay). These results support the temporal gradient view of retroactive interference (e.g. Wixted 2005), assuming that newly acquired memories are prone to interference immediately after learning/recall, as they continue to be processed ‘off-line’ during consolidation (Robertson 2012). Over time, these memories stabilise and become less susceptible to interference (Jost 1897; Müller and Pilzecker 1900; see also Mercer 2015).
5. *Mind*-*wandering during waking rest*. Craig et al. (2014) showed in healthy young adults that, compared to a waking rest condition, the retention of a word list was lower when learning was followed by novel picture encoding and autobiographical retrieval/future imagination, cued by concrete sounds (see also Varma et al. 2018). These results indicate that rich autobiographical thinking induced by external concrete cues during a waking rest phase can have detrimental effects on memory consolidation by transforming a resting condition into a distractor condition.
6. *Individual differences in immediate memory performance.* Martini et al. (2019a) showed a resting effect in children classified as lower-immediate memory performers, but not in children classified as higher- and middle-immediate memory performers. More specifically, 7-day delayed memory performance was higher in the waking rest condition compared to a visuospatial problem-solving condition in children with lower-immediate memory performance of a word list. Martini et al. (2019a, b) argued that children in the lower memory group probably built less stable memory representations, which were more affected by interference immediately after encoding.
7. *Order of the waking rest condition.* In a study by Martini et al. (2018b) with healthy older adults (60+ years) in which each participant was involved in a waking rest condition and a cognitive task condition (within-subject design), a beneficial effect of waking rest on delayed memory performance was found only when the cognitive task condition was followed by the waking rest condition, but not when the waking rest condition was followed by the cognitive task condition. One assumption of the authors was that the cognitive task condition had an interfering effect on the previous waking rest condition. These results suggest that, in older adults, the length and time of the waking rest phase becomes a relevant factor for memory with increasing age.
8. *Active recall.* Martini et al. (2018a) found a beneficial effect of waking rest after learning compared to solving visuospatial problems on 7-day recall performance of word lists, only when the learned information was reproduced in intermediary recall (in between immediate recall and 7-day recall; Experiment 1). No differences between the waking rest and visuospatial problem-solving condition on 7-day recall performance were found when the intermediary recall was omitted (Experiment 2). These results suggest that waking rest in combination with a subsequent active recall can have supporting effects on memory consolidation processes (Antony et al. 2017; Roediger and Karpicke 2006).

The proposed modulating factors have to be verified in further studies. Study results showing no difference between the impact of waking rest after learning and performing a cognitive task after learning should be integrated into the discussion about the resting effect, as they provide valuable additional information regarding under which conditions wakeful resting after learning is necessary and reasonable. In the context of modulating factors, it is essential to define what (wakeful) resting exactly means. Can we speak of waking rest when participants talk to the experimenter, read a newspaper article, hear music or lie in a noisy magnetic resonance scanner (for a review, see e.g. Dewar et al. 2007)? The modulating factors presented above are based on studies that defined resting as a state of minimal sensory and task-related cognitive input immediately after learning, where participants closed their eyes and relaxed during the waking rest phase for several minutes. However, additional modulating factors for the resting effect can be derived from studies that do not explicitly differentiate between a waking rest phase after learning and a task-related cognition phase.

For instance, study results indicate that the time course of memory consolidation is accelerated by prior knowledge relevant to the newly learned information (Dudai et al. 2015; Squire et al. 2015; van Kesteren et al. 2012). Together with the view that ‘more’ consolidated memories are less affected by subsequent interference (e.g. Robertson 2012), one can hypothesise that ‘prior knowledge’ can reduce the detrimental effects of task-related cognition after learning on memory, which should result in similar effects on memory between a waking rest condition and a task-related distraction condition after learning. Another example is (emotional) arousal. Evidence exists that arousal subsequent to learning affects memory consolidation (Mather and Sutherland 2011; McGaugh 2015). For instance, studies suggest that arousing stimuli subsequent to learning neutral stimuli can have detrimental but also enhancing effects on memory (see, e.g. Knight and Mather 2009). Accordingly, it can be hypothesised that, during a waking rest phase and—potentially independent from that, in a task-related cognition phase—arousal modulates the impact of the post-learning manipulation on memory consolidation. This speculative view suggests that not only the content of the specific post-learning condition, e.g. whether a content is verbal or visuospatial (see Dewar et al. 2007) in addition, the basic (emotional) arousal level (McGaugh 2015) during the respective post-learning condition, e.g. when participants search for errors in pictures or think about specific autobiographical content, modulates the resting effect.

To conclude, we presented a list of factors potentially modulating the impact of waking rest on memory, which have to be verified in further studies and additional factors have to be tested. In our view, it is vital to specify under which circumstances the resting effect occurs. Identification of such modulating factors can help in the design of future waking rest studies and interpretation of inconsistent findings; it also may serve as a basis for delineating practical implications on how to incorporate waking rest as a supporting memory retention strategy in everyday life.
